# Retraction: Hydrodynamic cavitation: a novel non-thermal liquid food processing technology

**DOI:** 10.3389/fnut.2024.1446767

**Published:** 2024-06-14

**Authors:** Frontiers Editorial Office

The Journal retracts the 2022 article cited above.

Following publication, the publisher found evidence of undisclosed conflicts of interest that undermined the integrity of the peer review process. As the scientific integrity of the article cannot be guaranteed, and in adherence to the recommendations of the Committee on Publication Ethics (COPE), the article is retracted.

This retraction was approved by the Chief Executive Editor of Frontiers. The authors received a communication regarding the retraction and had a chance to respond. This communication has been recorded by the publisher.

